# A Habit of Sleep

**Published:** 1881-10

**Authors:** 


### A HABIT OF SLEEP.

The best promoter of sleep is habit. It greatly helps the performance of the initial act, and a cultivation of the habit of going to sleep in a particular way, at a particular time, will do more to procure regular and healthy sleep than any other artifice. The artifice. The formation of the habit is, in fact, the creation or development of a special centre or combination in the nervous system, which will henceforward produce sleep as a natural rhythmical process. If this were more generally recognized, persons who suffer from sleeplessness of the sort which consists in simply being "unable to go to sleep," would set themselves resolutely to form such a habit. It is necessary that the training should be explicit and include attention to details. It is not very important what a person does with intention of going to sleep, but he should do precisely the same thing, in the same way, at the same time, until the habit is established.

A PERFECTLY square man is 'round at the right time.—*Whitehall Times.*

THE new road to San Francisco through Arizona runs for sixty miles along a basin that is 250 feet below the level of the ocean. This region has two to four feet of salt and alkali covering its surface. The ground is perfectly white.

